# Phase I-II study of hypofractionated simultaneous integrated boost using volumetric modulated arc therapy for adjuvant radiation therapy in breast cancer patients: a report of feasibility and early toxicity results in the first 50 treatments

**DOI:** 10.1186/1748-717X-7-145

**Published:** 2012-08-28

**Authors:** Marta Scorsetti, Filippo Alongi, Antonella Fogliata, Sara Pentimalli, Pierina Navarria, Francesca Lobefalo, Carlos Garcia-Etienne, Alessandro Clivio, Luca Cozzi, Pietro Mancosu, Giorgia Nicolini, Eugenio Vanetti, Marco Eboli, Carlo Rossetti, Arianna Rubino, Andrea Sagona, Stefano Arcangeli, Wolfgang Gatzemeier, Giovanna Masci, Rosalba Torrisi, Alberto Testori, Marco Alloisio, Armando Santoro, Corrado Tinterri

**Affiliations:** 1 Radiotherapy and radiosurgery, Humanitas Cancer Center, Istituto Clinico Humanitas, Rozzano, Milano, Italy; 2 Oncology Institute of Southern Switzerland, Medical Physics Unit, Bellinzona, Switzerland; 3 Breast Surgery, Humanitas Cancer Center, Istituto Clinico Humanitas, Rozzano, Milano, Italy; 4 Medical Oncology, Humanitas Cancer Center, Istituto Clinico Humanitas, Rozzano, Milano, Italy; 5 Thoracic Surgery, Humanitas Cancer Center, Istituto Clinico Humanitas, Rozzano, Milano, Italy

**Keywords:** Breast cancer, Simultaneous integrated boost, Hypofractionation, Volumetric modulated arc therapy

## Abstract

**Background:**

To report results in terms of feasibility and early toxicity of hypofractionated simultaneous integrated boost (SIB) approach with Volumetric Modulated Arc Therapy (VMAT) as adjuvant treatment after breast-conserving surgery.

**Methods:**

Between September 2010 and May 2011, 50 consecutive patients presenting early-stage breast cancer were submitted to adjuvant radiotherapy with SIB-VMAT approach using RapidArc in our Institution (Istituto Clinico Humanitas ICH). Three out of 50 patients were irradiated bilaterally (53 tumours in 50 patients). All patients were enrolled in a phase I-II trial approved by the ICH ethical committee. All 50 patients enrolled in the study underwent VMAT-SIB technique to irradiate the whole breast with concomitant boost irradiation of the tumor bed. Doses to whole breast and surgical bed were 40.5 Gy and 48 Gy respectively, delivered in 15 fractions over 3 weeks. Skin toxicities were recorded during and after treatment according to RTOG acute radiation morbidity scoring criteria with a median follow-up of 12 months (range 8–16). Cosmetic outcomes were assessed as excellent/good or fair/poor.

**Results:**

The median age of the population was 68 years (range 36–88). According to AJCC staging system, 38 breast lesions were classified as pT1, and 15 as pT2; 49 cases were assessed as N0 and 4 as N1. The maximum acute skin toxicity by the end of treatment was Grade 0 in 20/50 patients, Grade 1 in 32/50, Grade 2 in 0 and Grade 3 in 1/50 (one of the 3 cases of bilateral breast irradiation). No Grade 4 toxicities were observed. All Grade 1 toxicities had resolved within 3 weeks. No significant differences in cosmetic scores on baseline assessment vs. 3 months and 6 months after the treatment were observed: all patients were scored as excellent/good (50/50) compared with baseline; no fair/poor judgment was recorded. No other toxicities or local failures were recorded during follow-up.

**Conclusions:**

The 3-week course of postoperative radiation using VMAT with SIB showed to be feasible and was associated with acceptable acute skin toxicity profile. Long-term follow-up data are needed to assess late toxicity and clinical outcomes.

## Background

Breast-conserving surgery (BCS) with subsequent whole breast irradiation (WBI) is considered the standard of care for the majority of cases with early-stage breast carcinoma [1]. Mastectomy is reserved for patients ineligible for BCS due to clinical or technical surgical contraindications, or based on patient’s preference. Radiotherapy is delivered within 2–4 weeks after BCS, excluding patients receiving chemotherapy where radiotherapy starts usually within 3–4 weeks after the last cycle. Conventional radiotherapy after BCS is represented by WBI generally using two tangential fields for doses of 45–50 Gy/ 1.8–2 Gy per fraction. A boost irradiation with electron or photon beams to deliver a total tumour bed dose of 60–66 Gy is usually prescribed [2].

Although it is still not possible to definitively demonstrate the real radio sensitivity of breast cancer, the α/β of breast cancer is estimated to be around 4 [3]. This assumption suggests that hypofractionated regimens should be more effective than conventional fractionation. Moreover, to enforce the radiobiological rationale of hypofractionation in breast cancer is the shorter treatment time from 6 weeks to 3 weeks: the shorter the total treatment time, the lower the potential of repopulation of cancer cells, thus improving local control [3]. Starting from this radiobiological background, over the last 20 years, several randomized trials involving more than 7,000 women compared hypofractionated adjuvant radiotherapy to a standard regimen of 50 Gy in 25 fractions: START-A trial [4], START-B trial [5], RMH/GOC trial [6], ONTARIO trial [7]. Long-term results of these trials indicated similar rates of loco-regional relapse comparing the two radiation treatment arms. Evaluation of breast cosmesis at a median follow-up greater than 10 years was equivalent in both treatment arms [7]. It was largely confirmed that a 13–16 fraction regimen delivered over 3–4 weeks is as safe and effective as 50 Gy in 25 fractions. However, there is limited evidence from prospective randomized trials about the tolerability and efficacy of the tumour bed boost after hypofractionated WBI [8].

RapidArc® (Varian, Palo Alto, California, USA) is a relatively recently introduced volumetric modulated arc therapy (VMAT) technique based on simultaneous optimisation of multi leaf collimator (MLC) shapes, dose rate and gantry rotation speed [9]. The technology was investigated in several studies on different sites [10-13], showing a general improvement in sparing of organs at risk and healthy tissue, comparable target coverage, reduced beam-on time and lower number of monitor units (MU) compared to other intensity modulated radiotherapy (IMRT) approaches. RapidArc was introduced in our clinical practice since October 2009.

We present our clinical experience using VMAT for hypofractionated irradiation of the breast with simultaneous integrated boost (SIB) to the tumour bed.

## Methods

Fifty patients presenting early-stage breast carcinoma were enrolled at Humanitas Cancer Center of the Istituto Clinico Humanitas (Rozzano-Milan, Italy) between September 2010 and May 2011, in an institutional phase I-II prospective non-randomized trial of adjuvant radiotherapy with simultaneous integrated boost (SIB) delivered with RapidArc technology. The study was approved by the internal ethical committee and patient consent was obtained. The study will include 200 patients with a maximum period of enrolment of 48 months and a total period of duration of 10 years of follow-up. Primary endpoint of the study is to evaluate the feasibility of VMAT and hypofractionation with simultaneous integrated boost in breast cancer patients at early stage and undergoing conservative surgery. The feasibility is estimated in terms of: a) respecting of dose coverage for target volumes; b) respecting of dose tolerance levels for critical structures sparing (skin, heart, lungs, ribs). Secondary endpoint of the study is the evaluation of toxicity in terms of acute and late side effects. It will also be assessed the local control, even if it is not an explicit objective of the study. Grade 3–4 rate of toxicity is expected in nearly 1% of the cases. If, during the study, a Grade 3-4- toxicity incidence superior or equal to 5% is found, the protocol will be interrupted.

The study is still recruiting patients: here we present the preliminary data of feasibility and early toxicity of the first 50 patients.

Three out of 50 patients were irradiated for bilateral breast cancer. Eligibility criteria were: age >18 years, invasive cancer, American Joint Committee on Cancer AJCC Stage I to II, breast-conserving surgery, and any systemic therapy.

Radiotherapy treatment was started within 60 days from the surgical intervention; if adjuvant chemotherapy was administered, radiotherapy was started after 4 weeks from the last chemotherapy cycle. Patients received SIB irradiation of the whole breast and surgical bed at two different dose levels.

Clinical target volume of the whole breast (CTV_WB_) was the entire mammary gland. CTV for boost (CTV_boost_) was the surgical bed, as defined by surgical clips placed in the lumpectomy cavity during surgery. Planning target volumes (PTV) were contoured by adding a 5 mm margin to the CTV; PTV was limited to 4 mm within the skin surface, and excluded ribs and lung parenchyma. PTV_WB_ excluded the PTV_boost._.

Dose prescription was 40.5 Gy to PTV_WB_ and 48.0 Gy to PTV_boost_ in 15 fractions over 3 weeks, with simultaneous integrated boost delivering 2.7 and 3.2 Gy/fraction for each PTV respectively [14].

Plan objectives were the following:

Target coverage and homogeneity: D_98%_ > 95% for both PTVs, and D_2%_ < 107% for the high dose PTV (each percentage dose value is relative to its specific target). Concerning organs at risk: ipsilateral lung receiving mean dose less than 10 Gy, and the volume receiving more than 20 Gy not exceeding 10% (V_20Gy_ < 10%) [14]; heart volume receiving more than 40 Gy not exceeding 3% and 18 Gy not exceeding 5% [14]; minimize contralateral lung and breast irradiation; ribs maximum dose not exceeding 50 Gy; skin dose not exceeding 40–45 Gy for cutaneous desquamation: skin dose was recorded for 3 and 5 mm thickness of the first skin layers in a region covering the whole breast plus an additional margin of 3 cm around the mammary gland. Healthy tissue, defined as the acquired CT dataset subtracting the PTVs, was also analysed.

Patients were in supine position, with both arms above the head. CT dataset was acquired with 3 mm thick adjacent slices. No respiratory gating was adopted. Plans were optimized for VMAT treatments with two partial arcs in a range from the classical medial tangential and the posterior entrances through the PTV side; Progressive Resolution Optimizer was used to modulate MLC shape and beam intensity during the gantry rotation. Delivery was on a 6MV beam from a Clinac DHX equipped with a Millennium MLC-120. Dose calculations used the Anisotropic Analytical Algorithm (AAA). Daily cone beam CT (CBCT) images were generated before each treatment session in each patient to verify the set-up. To minimize patient positioning errors, after automatic co-registration of CBCT and simulation CT images, corrections were manually done daily by operators.

Median follow-up was 12 months (range 8–16). Follow-up was scheduled according to our internal guidelines at the end of radiotherapy, at 3 and 6 months after radiation treatment, and then every 6 months for the first 3 years. Hematologic studies, as well as bilateral mammography and breast ultrasound were scheduled every 12 months. Skin toxicity was visually assessed by objective clinical exam and photography of irradiated breast in frontal and lateral view during each visit (during treatment and during follow-up). Pictures were compared with baseline performed before the beginning of the radiation treatment, and toxicity was scored according to RTOG acute radiation morbidity scoring criteria. Cosmetic outcomes were ranked as: excellent/good vs. fair/poor [15].

Dosimetric evaluation was based on DVH analysis of targets and organs at risk. Data were reported as mean doses, V_x_ (volume receiving more than x dose) and D_y_ (dose received by at least y volume). For cases with bilateral disease, the dosimetric assessment was considered for each breast (total of 53 cases), and data refer to the plan sum for ipsilateral structures (lung, breast), while contralateral structures were excluded from the analysis.

## Results

Patient and tumour characteristics are shown in Table 1. Median age of the population under investigation was 68 years (range 36–88). According to AJCC staging system, 38 breast lesions were classified as pT1, and 15 as pT2; 49 cases were assessed as pN0 and 4 as pN1.

**Table 1:** Patient, tumour characteristics and disease stages for 53 breast cancers on 50 patients

**Age** [years]	**Median**	**68**
	**Range**	**36-88**
T	pT1mi	1 (2%)
	pT1a	6 (11%)
	pT1b	11 (21%)
	pT1c	20 (38%)
	pT2	15 (28%)
N	pN0	49 (92%)
	pN1	4 (8%)
Histology	IDC	41 (77%)
	ILC	10 (19%)
	Other Invasive	2 (4%)
	DCIS	0 (0%)
Grading	I	10 (19%)
	II	38 (72%)
	III	5 (9%)
Estrogen Receptors	Positive	49 (92%)
	Negative	4 (8%)
Progesterone Receptors	Positive	46 (87%)
	Negative	7 (13%)
Her-2/neu	Overexpressed	1 (2%)
	Not overexp.	52 (98%)
Ki-67	≥20	3 (6%)
	<20	50 (94%)
Disease Stage	0	0 (0%)
	I	43 (81%)
	II	10 (19%)

IDC: invasive ductal carcinoma; ILC: invasive lobular carcinoma; DCIS: ductal carcinoma *in situ.*

Clinical results are summarized in Table 2. The maximum acute skin toxicity by the end of treatment was Grade 0 in 20/50 (40%) patients, Grade 1 in 32/50 (64%), Grade 2 in 0 and Grade 3 in 1/50 (2%) (one of the 3 cases of bilateral breast irradiation). No Grade 4 toxicities were observed. After radiation, all Grade 1 toxicities had resolved within 3 weeks. There were no significant differences in cosmetic scores from baseline assessment vs. 3 and 6 months after the treatment: all patients were scored as excellent/good cosmesis (50/50) compared with baseline. No fair/poor judgment was recorded in the 50 patients during follow-up. No other toxicities or local failures were recorded during follow-up.

**Table 2:** Description of acute skin toxicity in the population of study, stratified for each grade, according to RTOG scale and clinical results

**Grade**	**Skin toxicity description**	**Number of cases**	**% of cases**
Grade 0	No change over baseline	20/50	40%
Grade 1	Follicular, faint or dull erythema/ epilation/dry desquamation/ decreased sweating	32/50	64%
Grade 2	Tender or bright erythema, patchy moist desquamation/ moderate edema	0/50	0%
Grade 3	Confluent, moist desquamatiom other than skin folds, pitting edema	1/50	2%
Grade 4	Ulceration, hemorrhage, necrosis	0/50	0%

**Figure 1:**
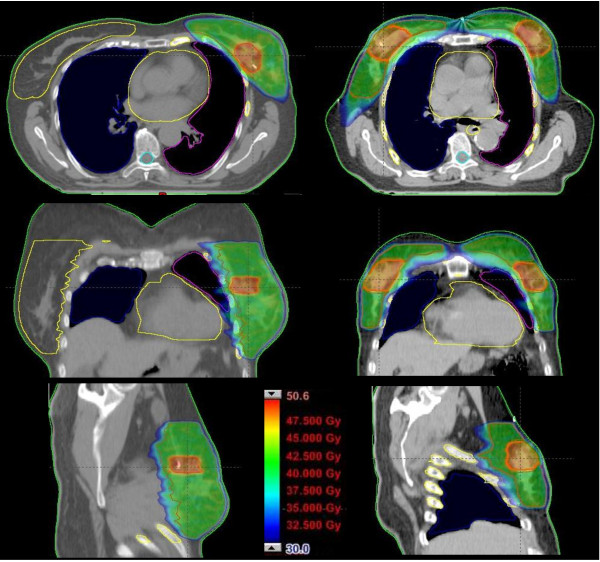
Dose distributions in on axial, coronal and sagittal slices for a left side breast case (on the right) and for a bilateral case (on the left).

**Figure 2:**
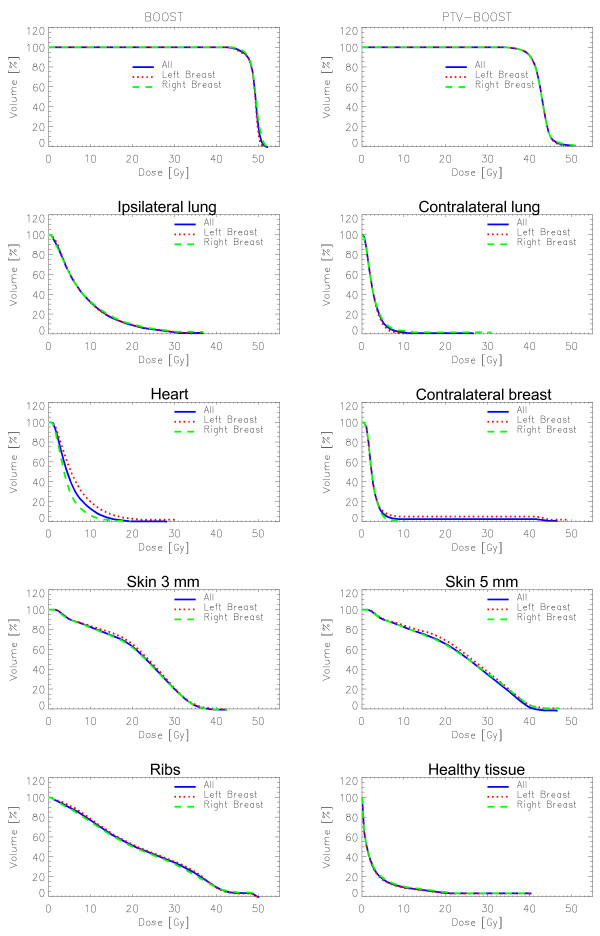
Average Dose-volume histograms DVH for targets and critical structures, stratified as left, right side breast and all cases together.

Dosimetric results are summarized in Figure 1 where a qualitative dose distribution for a single patient is shown, and in Figure 2, where the average DVHs for targets and organs at risk is presented. Main statistic parameters are reported in Table 3 as mean values and standard deviation over all patients, for each parameter. Results are stratified according to side (left or right) and also presented all together.

**Table 3:** Dosimetric results

	**Parameter**	**All**	**Left breast**	**Right breast**
PTV_WB_	Volume [cm^3^]	605.6 ± 313.9	702.5 ± 291.7	505.1 ± 309.5
	Mean [Gy]	41.5 ± 1.3	41.5 ± 1.3	41.5 ± 1.4
	D_2%_ [Gy]	45.0 ± 1.3	44.8 ± 1.1	45.1 ± 1.5
	D_98%_ [Gy]	37.2 ± 2.9	37.2 ± 3.1	37.3 ± 2.8
	V_95%_ [%]	92.9 ± 12.8	93.0 ± 13.3	92.8 ± 12.6
	V_105%_ [%]	30.8 ± 16.8	30.9 ± 16.0	30.7 ± 18.0
PTV_boost_	Volume [cm^3^]	51.5 ± 45.9	54.11 ± 50.3	48.8 ± 41.7
	Mean [Gy]	47.8 ± 1.0	47.7 ± 1.1	48.0 ± 0.9
	D_2%_ [Gy]	49.3 ± 0.9	49.1 ± 0.9	49.5 ± 0.9
	D_98%_ [Gy]	45.8 ± 1.4	45.7 ± 1.6	45.8 ± 1.3
	V_95%_ [%]	93.5 ± 19.9	92.5 ± 23.7	94.6 ± 15.4
	V_105%_ [%]	0.7 ± 2.1	0.2 ± 0.7	1.2 ± 2.9
Ipsilateral Lung	Mean [Gy]	8.7 ± 1.7	8.5 ± 1.7	8.9 ± 1.8
	V_5Gy_ [%]	61.9 ± 15.9	60.0 ± 16.1	63.9 ± 15.9
	V_20Gy_ [%]	8.6 ± 2.9	8.4 ± 2.8	8.8 ± 3.0
	V_25Gy_ [%]	4.1 ± 1.9	4.2 ± 1.9	3.9 ± 1.9
Contralateral Lung	Mean [Gy]	2.5 ± 0.9	2.6 ± 0.9	2.5 ± 1.0
	V_5Gy_ [%]	8.9 ± 9.9	8.8 ± 9.7	9.1 ± 10.4
Heart	Mean [Gy]	5.4 ± 2.0	6.5 ± 1.7	4.3 ± 1.8
	D_1cm3_ [Gy]	20.4 ± 8.9	27.0 ± 4.4	13.1 ± 6.4
	V_18Gy_ [%]	2.0 ± 2.2	3.1 ± 3.3	0.5 ± 1.2
Skin 3 mm	Mean [Gy]	21.0 ± 1.6	21.4 ± 1.8	20.7 ± 1.4
	V_30Gy_ [cm^3^]	27.0 ± 12.5	29.6 ± 14.2	24.2 ± 9.9
	V_40Gy_ [cm^3^]	0.5 ± 0.9	0.6 ± 1.1	0.5 ± 0.7
Skin 5 mm	Mean [Gy]	23.3 ± 1.7	23.6 ± 1.8	22.9 ± 1.4
	V_30Gy_ [cm^3^]	88.3 ± 26.7	96.0 ± 28.5	80.3 ± 22.6
	V_40Gy_ [cm^3^]	5.9 ± 5.6	6.1 ± 6.5	5.6 ± 4.6
Ribs	D_1cm3_ [Gy]	37.4 ± 2.6	37.4 ± 2.7	37.5 ± 2.5
	V_40Gy_ [cm^3^]	3.2 ± 3.6	3.2 ± 3.9	3.2 ± 3.4
Contralateral Breast	Mean [Gy]	3.3 ± 5.8	4.1 ± 8.2	2.5 ± 0.6
	V_5Gy_ [%]	7.6 ± 15.0	10.0 ± 20.3	5.1 ± 4.8
Healthy Tissue	Mean [Gy]	3.4 ± 0.7	3.3 ± 0.6	3.5 ± 0.7
	V_10Gy_ [%]	9.2 ± 2.2	8.8 ± 1.9	9.6 ± 2.3
	DoseInt [Gy*cm^3^*10^4^]^*^	7.2 ± 2.0	7.6 ± 1.9	6.8 ± 2.0

^*^ DoseInt = integral dose.

Concerning target coverage, the PTV_boost_ inhomogeneity was in average well within the requirements of ±5% dose variation; while for PTV_WB_ the minimum significant dose (D_98%_) was in average 92%, and the maximum significant dose (D_2%_) was in average 111% of the whole breast dose prescription; such high doses were accepted, being located in proximity of the boost region, where the gradient between the two dose levels of the simultaneous integrated boost is located and unavoidable; for the same reason the mean PTV_WB_ dose was 2.4% higher than the prescription.

Dose to lungs was kept within tolerance levels, in particular the mean lung dose of the ipsilateral breast was in average lower than 9 Gy. Heart did not receive high doses, with no volume at 40 Gy dose level, and with a maximum significant dose (D_1cm3_) of 27 Gy for left breast lesions. Skin dose level, thanks to the build-up effect, was between 21 and 23 Gy in average, considering a 3 or 5 mm thick superficial layer. Whole dose of 40 Gy started to be delivered at a depth of about 5 mm or more. Ribs volume did not exceed 50 Gy, having a maximum significant (D_1cm3_) dose of about 37 Gy. For the contralateral breast it should be noted that the mean dose (well below 5 Gy in average) was higher for left side lesions, where the optimizer was forced to lower the heart dose, distributing the surrounding dose to other locations, including the right breast.

No correlation (ANOVA analysis) was found between skin toxicity and skin dose (mean dose to 3 mm thick skin was 21.0 ± 1.6 Gy for G0 and 21.2 ± 1.5 Gy for G1 groups), nor with skin structure volume (mean volume of 3 mm thick skin was 159 ± 39 cm^3^ for G0 and 152 ± 35 cm^3^ for G1 groups).

Also chemotherapy showed no correlations to skin toxicity, and all 5 patients who underwent chemotherapy had G0 skin toxicity.

## Discussion

Dose per fraction size in adjuvant irradiation of the breast is expected to significantly impact on the clinical results: fractionation sensitivity of breast cancer cell seems to be rather high, similar to that of late-reacting normal tissues. In fact, data from four randomized trials with early-stage breast cancer patients [4-7] support the hypothesis that hypofractionation with a modest increase in dose per fraction, accompanied by a modest decrease in total dose, is likely to result in similar outcomes compared with conventional fractionation with respect to local control and late radiation toxicity. Thus, on the basis of level I evidence from these clinical trials, this seems to be solid evidence to prescribe modest hypofractionation for the adjuvant treatment of women requiring WBI [4-7]. Conversely, an object of debate remains the role of the boost to the surgical bed, especially when hypofractionation is the selected schedule for whole breast irradiation. Recently, an ASTRO task force to define guidelines for breast hypofractionation [8] stated that, when a boost is indicated, there was a lack of consensus regarding the appropriateness of hypofractionation for whole breast irradiation. In the Canadian trial [7], none of the patients received a boost, but the risk of local relapse at 10 years was only 7.5%, suggesting that the influence of a tumor bed boost could be really limited. In START A and B trials [4,5], less than 50% of patients received a boost after whole breast irradiation, but further specific results of this subgroup have not been published. The RMH/GOC trial included 723 patients randomized to receive or not a boost to the tumor bed; however, data on local relapse by subgroups were not reported in that study and it is not possible to clearly define whether or not a boost adds a benefit to hypofractionated WBI.

In absence of any type of evidence concerning boost, the ASTRO task force does not suggest a specific schedule for the tumor bed when given in conjunction with hypofractionated WBI. Recent retrospective data suggest that patients with known negative margins have high local control rates when boost was not prescribed following WBI [16]. Thus, based on published reports available in literature, boost doses of 10–16 Gy in 2 Gy/fraction or 10 Gy in 2.5 Gy/fraction were considered acceptable [8,17]. In the present study, boost doses were clearly higher than those suggested by the ASTRO panel. However, the studies considered by this panel included patients treated with bi-dimensional or three-dimensional conformal radiotherapy. From radiobiological point of view, an α/β ratio of 4 Gy (4) has been confirmed by the results of START trials [4] and [5]. Consequently, for breast tumours, the biologically effective dose (BED) should be strictly related to high fractionation sensitivity and BED-response relationship could be more clinically impacting than the conventional dose–response relationship. In a deep analysis published by Plataniotis et al. on the radiobiological issue of the dose–response, a linear regression equation linking BED to Tumour control Probability (TCP) was derived[18]. A TCP >90% for BEDs >90 Gy_4_ (calculated using α/β = 4 Gy for breast cancer cells) has been also estimated. The BED of whole breast RT of 50 Gy/25 fractions is 75 Gy_4_, and where a boost of 10 Gy is added, the total BED rises to 90 Gy_4_. With the regimen of fractionation of the present study, using a concomitant boost for a total dose of 48 Gy in 15 sessions, the total BED estimated is 86 Gy_4._ Although this BED is slightly lower than 90 Gy_4_, it is superior than the BEDs of START trials doses, when frequently no sequential boost was added. Thus, starting from these considerations, the use of concomitant boost could be seriously considered in hypofractionated schedules to further increase the TCP.

The accuracy of using the scar to define the lumpectomy cavity for boosting has been questioned [19] and surgical clips can be considered as ideal for the correct identification of the tumor bed [20]. In our experience, due to the VMAT technique, we were able to focus the boost dose on the surgical bed defined on simulation CT with the help of surgical clips and deliver it concomitantly with the hypofractionated WBI.

Few experiences have been published with intensity modulated hypofractionation associated with surgical bed boost. A four-week course of radiation for breast cancer using hypofractionated IMRT with an incorporated boost in 75 patients was reported by Freedman et al. [21]. The whole breast received 45 Gy and the lumpectomy bed 56 Gy in 20 treatments over 4 weeks. In our protocol, compared to that four-week course study by Freedman, worse results could be expected for the reduced total treatment time by one week. On the contrary, in our study, the amount of acute Grade 1 events was the same (64% for both studies), the group of patients without side effects was greater (40% vs. 12%), and Grade 2 was substantially better (0% vs. 23%). Concerning Grade 3 events, the sole case in our study (no cases in Freedman study) referred to a bilateral breast irradiation.

An acute toxicity comparison between WBI using 3-week schedule with a concomitant boost and the 6.5-week conventional schedule with sequential boost was recently published [22]. Chadha et al., in the accelerated schedule with SIB prescribed similar doses of current study: 40.5 Gy in 2.7 Gy/fraction to the whole breast with 4.5 Gy in 0.3 Gy/fraction for the concomitant boost, delivering a total dose of 45.0 Gy in 3.0 Gy/fraction to the lumpectomy site. The study showed that no significant difference in the incidence of breast edema, fatigue, or hematologic side effects was observed between the 3-week and the 6.5-week groups. The 3-week arm presented Grade ≥ 2 skin toxicity in 4% of the patients, in line with our data and using similar doses.

The schedule of 3-week course with 40.5 Gy to whole breast and 48 Gy to lumpectomy site adopted in the present study was already published by Formenti et al [14]. The Grade 1–2 skin dermatitis reported by Formenti are similar to those report in the current study (67% vs. 64%) confirming the feasibility of such a fractionation.

Regarding dosimetry evaluation in our study, the protocol objectives were met. To note is the average skin dose in the first 3 mm of 21 Gy, delivered as 1.4 Gy/fraction, while in the conventional fractionation of a 50 Gy treatment, the skin dose would have been about 26 Gy, at only 1 Gy/fraction.

Symptomatic pneumonitis is usually infrequent after conventional breast irradiation. Given the larger V_5Gy_ value of VMAT plans compared with conventional irradiation, an assessment of pulmonary toxicity, regarding change in pulmonary function and radiologic findings was considered. However, the clinical respiratory syndrome is usually noted several months after irradiation and it could be definitively assessable only in a longer follow-up. We will report that data in a further study, focused on late toxicities in the same patient population.

In conclusion, the presented experience of a 3-week course of postoperative radiation using VMAT with SIB is feasible and it was associated with acceptable acute skin toxicity, similar to those recently reported in literature. Long-term follow-up data are needed to assess late toxicity and clinical outcomes.

## Competing interests

Dr. L. Cozzi acts as Scientific Advisor to Varian Medical Systems and is Head of Research and Technological Development to Oncology Institute of Southern Switzerland, IOSI, Bellinzona.

## Authors’ contributions

MS, FA, SP and PN coordinated the entire study. Data collection and clinical data analysis were conducted by CGE, ME, CR, AR, AS, SA, WG, GM, RT, AT, MA, AS, CT. Dosimetric data collection and analysis were conducted by AF, FL, AC, LC, PM, GN, EV. All authors read and approved the final manuscript.

## References

[B1] Veronesi U Mariani L Greco M Saccozzi R Luini A Aguilar M Marubini E Twenty year follow-up of a randomized study comparing breast-conserving surgery with radical mastectomy for early breast cancer N Engl J Med 2002 347 1227 1232 10.1056/NEJMoa020989 12393819

[B2] American College of Radiology Practice guideline for the breast conservation therapy in the management of invasive breast carcinoma J Am Coll Surg 2007 205 362 376 17660085 10.1016/j.jamcollsurg.2007.02.057

[B3] Qi XS White J Li XA Is α/β for breast cancer really low? Radiother Oncol 2011 100 282 288 10.1016/j.radonc.2011.01.010 21367477

[B4] Bentzen SM Agrawal RK Aird EG Barrett JM Barrett-Lee PJ Bliss JM Brown J Dewar JA Dobbs HJ Haviland JS Hoskin PJ Hopwood P Lawton PA Magee BJ Mills J Morgan DA Owen JR Simmons S Sumo G Sydenham MA Venables K Yarnold JR The UK Standardisation of Breast Radiotherapy (START) Trial A of radiotherapy hypofractionation for treatment of early breast cancer: A randomised trial Lancet Oncol 2008 9 331 341 18356109 10.1016/S1470-2045(08)70077-9 PMC2323709

[B5] Bentzen SM Agrawal RK Aird EG Barrett JM Barrett-Lee PJ Bentzen SM Bliss JM Brown J Dewar JA Dobbs HJ Haviland JS Hoskin PJ Hopwood P Lawton PA Magee BJ Mills J Morgan DA Owen JR Simmons S Sumo G Sydenham MA Venables K Yarnold JR The UK Standardisation of Breast Radiotherapy (START) Trial B of radiotherapy hypofractionation for treatment of early breast cancer: A randomised trial Lancet 2008 371 1098 1107 18355913 10.1016/S0140-6736(08)60348-7 PMC2277488

[B6] Owen JR Ashton A Bliss JM Homewood J Harper C Hanson J Haviland J Bentzen SM Yarnold JR Effect of radiotherapy fraction size on tumour control in patients with early-stage breast cancer after local tumour excision: long-term results of a randomised trial Lancet Oncol 2006 7 467 471 10.1016/S1470-2045(06)70699-4 16750496

[B7] Whelan TJ Pignol JP Levine MN Julian JA MacKenzie R Parpia S Shelley W Grimard L Bowen J Lukka H Perera F Fyles A Schneider K Gulavita S Freeman C Long-term results of hypofractionated radiation therapy for breast cancer N Engl J Med 2010 362 513 520 10.1056/NEJMoa0906260 20147717

[B8] Smith BD Bentzen SM Correa CR Hahn CA Hardenbergh PA Ibbott GS McCormick B McQueen JR Pierce LJ Powell SN Recht A Taghian AG Vicini FA White JR Haffty BG Fractionation for whole breast irradiation: an American Society for Radiation Oncology (ASTRO) evidence-based guideline Int J Radiat Oncol Biol Phys 2011 81 59 68 10.1016/j.ijrobp.2010.04.042 20638191

[B9] Otto K Volumetric modulated arc therapy: IMRT in a single arc Med Phys 2008 35 310 317 10.1118/1.2818738 18293586

[B10] Bignardi M Cozzi L Fogliata A Lattuada P Mancosu P Navarria P Urso G Vigorito S Scorsetti M Critical appraisal of volumetric modulated arc therapy in stereotactic body radiation therapy for metastases to abdominal lymph nodes Int J Radiat Oncol Biol Phys 2009 75 1570 1577 10.1016/j.ijrobp.2009.05.035 19880261

[B11] Clivio A Fogliata A Franzetti-Pellanda A Nicolini G Vanetti E Wyttenbach R Cozzi L Volumetric-modulated arc radiotherapy for carcinomas of the anal canal: A treatment planning comparison with fixed field IMRT Radiother Oncol 2009 92 118 124 10.1016/j.radonc.2008.12.020 19181409

[B12] Verbakel W Senan S Cuijpers JP Slotman BJ Lagerwaard FJ Rapid delivery of stereotactic radiotherapy for peripheral lung tumors using volumetric intensity-modulated arcs Radiother Oncol 2009 93 122 124 10.1016/j.radonc.2009.05.020 19552979

[B13] Vanetti E Clivio A Nicolini G Fogliata A Ghosh-Laskar S Agarwal JP Upreti RR Budrukkar A Murthy V Deshpande DD Shrivastava SK Dinshaw DA Cozzi L Volumetric modulated arc radiotherapy for carcinomas of the oro-pharynx, hypo-pharynx: A treatment planning comparison with fixed field IMRT Radiother Oncol 2009 92 111 117 10.1016/j.radonc.2008.12.008 19157609

[B14] Formenti SC Gidea-Addeo D Goldberg JK Roses DF Guth A Rosenstein BS DeWyngaert KJ Phase I-II Trial of Prone Accelerated Intensity Modulated Radiation Therapy to the Breast to Optimally Spare Normal Tissue J Clin Oncol 2007 25 2236 2242 10.1200/JCO.2006.09.1041 17470849

[B15] Harris JR Levene MB Svensson G Hellman S Analysis of cosmetic results following primary radiation therapy for stages I and II carcinoma of the breast Int J Radiat Oncol Biol Phys 1979 5 257 261 10.1016/0360-3016(79)90729-6 110740

[B16] Arthur DW Cuttino LW Neuschatz AC Koo DT Morris MM Bear HD Kaplan BJ Dawson K Wazer DE Tumor bed boost omission after negative re-excision in breast-conservation treatment Ann Surg Oncol 2006 13 794 801 10.1245/ASO.2006.04.002 16614879

[B17] Romestaing P Lehingue Y Carrie C Coquard R Montbarbon X Ardiet JM Mamelle N Gérard JP Role of a 10-Gy boost in the conservative treatment of early breast cancer: Results of a randomized clinical trial in Lyon, France J Clin Oncol 1997 15 963 968 9060534 10.1200/JCO.1997.15.3.963

[B18] Plataniotis A Dale RG Biologically Effective Dose–Response Relationship for Breast Cancer Treated by Conservative Surgery and Postoperative Radiotherapy Int J Radiat Oncol Biol Phys 2009 75 512 517 10.1016/j.ijrobp.2009.05.013 19625139

[B19] Oh KS Kong FM Griffith KA Yanke B Pierce LJ Planning the breast tumour bed boost: changes in the excision cavity volume and surgical scar location after breast-conserving surgery and whole-breast irradiation Int J Radiat Oncol Biol Phys 2006 66 680 686 10.1016/j.ijrobp.2006.04.042 16863683

[B20] Solin LJ Danoff BF Schwartz GF Galvin JM Goodman RL A practical technique for the localization of the tumour volume in definitive irradiation of the breast Int J Radiat Oncol Biol Phys 1985 11 1215 1220 10.1016/0360-3016(85)90072-0 3997603

[B21] Freedman GM Anderson PR Goldstein LJ Ma CM Li J Swaby RF Litwin S Watkins-Bruner D Sigurdson ER Morrow M Four-week course of radiation for breast cancer using hypofractionated intensity modulated radiation therapy with an incorporated boost Int J Radiat Oncol Biol Phys 2007 68 347 353 10.1016/j.ijrobp.2006.12.035 17379430

[B22] Chadha M Vongtama D Friedmann P Parris C Boolbol SK Woode R Harrison LB Comparative Acute Toxicity from Whole Breast Irradiation Using 3-Week Accelerated Schedule With Concomitant Boost and the 6.5-Week Conventional Schedule With Sequential Boost for Early-Stage Breast Cancer Clin Breast Cancer 2012 12 57 62 10.1016/j.clbc.2011.09.002 22056970

